# pH as a Design Tool for Low-Molecular-Weight Hydrogelators: Triggers, Structural Control, and Orthogonal Assembly

**DOI:** 10.3390/gels12040344

**Published:** 2026-04-20

**Authors:** Rie Kakehashi

**Affiliations:** Morinomiya Center, Osaka Research Institute of Industrial Science and Technology, 1-6-50 Morinomiya, Joto-ku, Osaka 536-8553, Japan; rie@orist.jp

**Keywords:** low-molecular-weight hydrogel, supramolecular gel, pH-responsive hydrogelator, N-oxide, boronic acid, self-assembly, orthogonal system

## Abstract

Low-molecular-weight gelators (LMWGs) have attracted growing attention as versatile alternatives to conventional polymeric thickeners and gelators, owing to their ability to form three-dimensional fibrillar networks through non-covalent self-assembly and to undergo reversible sol–gel transitions in response to external stimuli. Among the various stimuli that can be exploited, pH represents a particularly attractive trigger given its direct relevance to biological and physiological environments. This review focuses on three categories of pH-responsive LMWGs that have shown notable progress over the past decade yet remain relatively underexplored in the literature. First, N-oxide-type hydrogelators are discussed, with emphasis on amide amine oxide-based surfactants and pyridine-N-oxide frameworks. The pH-dependent protonation of the N-oxide moiety modulates intermolecular hydrogen bonding, thereby governing self-assembly and gel formation. The structural versatility of these gelators enables rational tuning of aggregate morphology and confers clear pH and temperature responsiveness. Second, recent advances in phenylboronic acid-based LMWGs are highlighted. Although boronic acid derivatives have long been studied as dynamic crosslinking units in polymeric hydrogels, 3-isobutoxyphenylboronic acid was recently identified as the first example of phenylboronic acid functioning as an LMWG, in which gelation is driven primarily by hydrogen bonding and pH responsiveness is exploited for stimuli-triggered gel disruption rather than gel formation. Third, pH-responsive orthogonal self-assembly systems are reviewed. Representative examples include multicomponent hybrid hydrogels combining pH-activated LMWGs with polymer gelators for controlled drug release, pH-triggered self-sorting of two LMWGs without any polymeric component, and bio-based orthogonal hydrogels composed of a glucolipid LMWG and cellulose nanocrystals. For each system, both advantages and remaining limitations are critically assessed. Collectively, this review aims to provide a timely overview of emerging trends in pH-responsive LMWG research and to offer perspectives on the rational design of next-generation stimuli-responsive soft materials.

## 1. Introduction

Rheological control of liquids has long been a critical challenge in industrial applications. Polymeric materials have traditionally been employed as thickeners and gelators. In recent years, however, low-molecular-weight gelators (supramolecular gelators), which induce thickening and gelation through the self-assembly of small molecules in liquids, have attracted considerable attention. It is generally understood that low-molecular-weight gelators (LMWGs) function by forming molecular aggregates in solution, which subsequently associate in a hierarchical manner to yield progressively larger assemblies, ultimately constructing a three-dimensional network that suppresses the fluidity of the liquid. Gels formed by LMWGs are physical gels devoid of covalent crosslinks, and they typically undergo reversible sol–gel transitions in response to temperature changes. In the case of polymeric thickeners and gelators, once dissolved in a solvent, the viscosity does not decrease appreciably even at elevated temperatures, making handling difficult. Low-molecular-weight gelators, by contrast, exhibit a dramatic reduction in viscosity above the gelation temperature (sol–gel transition temperature), which renders them considerably easier to handle.

This constitutes a notable advantage of LMWGs. On the other hand, the mechanical strength of low-molecular-weight gels is substantially inferior to that of chemically crosslinked polymer gels, which represents a significant drawback. This weakness stems not merely from the absence of fixed crosslinking points, but more fundamentally from the non-covalent nature of the intermolecular interactions—such as hydrogen bonding, hydrophobic interactions, and π–π stacking—that drive self-assembly. Because these interactions are inherently weaker and more dynamic than covalent bonds, the resulting fibrillar networks are susceptible to disruption under mechanical stress, even when an apparently continuous three-dimensional network has been formed. In review [1], a detailed comparison between polymeric gelators and low-molecular-weight gelators is provided, and readers are strongly encouraged to consult this reference.

The self-assembly of low-molecular-weight compounds is governed by a variety of intermolecular interactions, including hydrophobic interactions, π–π interactions, electrostatic interactions, and hydrogen bonding. Consequently, the gel properties are strongly influenced by how these interactions are reflected in the molecular structure of the low-molecular-weight gelator. While the broad range of available chemical structures offers the advantage of considerable freedom in molecular design, this same diversity of options can conversely pose a significant challenge when attempting to design molecules that exhibit the desired gel properties.

Control over the self-assembled structures formed through the self-assembly of low-molecular-weight compounds is a topic of considerable interest to researchers in the fields of colloid and interface science as well as supramolecular chemistry, and a large number of studies have been reported to date. Since the 1990s, low-molecular-weight hydrogelators (LMWHGs) driven by hydrophobic interactions, van der Waals forces, hydrogen bonding, and other non-covalent interactions have been covered in numerous reviews; here, however, we restrict our coverage to those published over approximately the past decade [2,3,4,5,6,7,8,9,10,11,12,13,14,15,16,17,18,19,20,21,22,23,24,25,26]. These include works addressing supramolecular gels broadly [2,3,4,10,15,16,17,23], those focused primarily on diverse applications such as sensors, optical materials, energy conversion, and medical uses [10,13,14,15,18,19,20,21,24,25], hydrogen-bonding-driven systems [9], supramolecular gels with steroid scaffolds [6], gels based on sugars [11,26], amino acids and peptides [5,8,14], urea derivatives [7], surfactant-based systems [12], and surfactant-mediated orthogonal hydrogels [22]. Among these, a review by Smith et al. published in 2024 [23] covers an exceptionally wide range of low-molecular-weight gelators and serves as an invaluable resource for gaining a broad overview of the field.

This review focuses on several pH-responsive LMWHGs that have shown notable progress over the past decade, yet remain relatively underexplored in the literature—specifically, N-oxide-type LMWHGs and phenylboronic acid-type LMWHGs. In addition, we introduce recent advances in orthogonal self-assembly, a strategy in which multiple components independently form distinct supramolecular aggregates in aqueous media. First, N-oxide-type LMWHGs represent one class of pH-responsive LMWGs [27,28,29,30,31,32,33,34,35]. Representative examples of pH-responsive LMWGs include fatty acid soaps [36,37,38,39] and amino acid derivatives [40,41,42,43,44,45,46,47,48,49,50]. In many of these systems, ionic groups are dissociated at high pH, whereas gelation occurs at low pH through suppression of intermolecular electrostatic repulsion, which promotes self-assembly. The N-oxide group, although a weak electrolyte, is characterized by its high polarity, which confers high water solubility across a wide pH range [51,52]. At high pH, the N-oxide group is nonionic and does not interfere with self-assembly, whereas at low pH it becomes cationic upon protonation, suggesting potential antimicrobial activity. Next, we introduce recent examples of stimuli-responsive gels in which phenylboronic acid itself acts as an LMWG [53]. Conventionally, boronic acids and boronic acid derivatives have been extensively studied as dynamic crosslinking units incorporated into polymers, owing to the significant contribution of such dynamic crosslinks to the self-healing properties of the resulting gels [54,55,56,57,58,59,60,61,62,63,64,65,66,67,68,69,70,71]. Recently, however, boronic acid derivatives that function as low-molecular-weight gelators have been identified [53]. Finally, we discuss orthogonal self-assembly. In pH-responsive orthogonal systems, at least one component undergoes self-assembly or disassembly in response to pH [72,73,74,75]. This pH responsiveness may manifest in different ways: in some systems, a change in pH serves directly as the gelation trigger, whereas in others, pH acts not to induce gelation but rather to destabilize the gel network, thereby triggering the release of encapsulated drugs.

**Figure 1:**
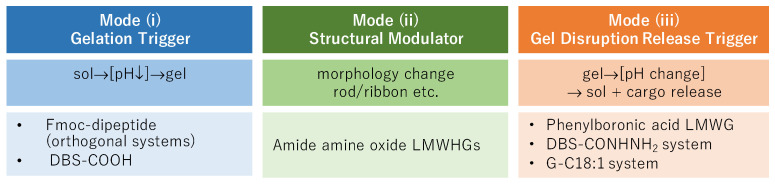
Role of pH in LMWG self-assembly in this review.

Although all three classes discussed in this review are described as pH-responsive, the role that pH plays differs fundamentally across systems and can be classified into three distinct modes (Figure 1): (i) pH as a gelation trigger, in which a change in pH directly initiates self-assembly and gel formation; (ii) pH as a structural modulator, in which pH governs the morphology of self-assembled aggregates without necessarily acting as the primary gelation switch; and (iii) pH as a gel-disruption trigger, in which pH does not induce gelation but instead destabilizes the assembled network, thereby enabling stimuli-responsive release of encapsulated molecules. In amide amine oxide-based LMWHGs, pH controls the degree of protonation of the N-oxide group and thereby modulates aggregate morphology, exemplifying mode (ii). In phenylboronic acid-based LMWGs, pH acts as a disruption trigger for a gel whose formation is driven primarily by hydrogen bonding, exemplifying mode (iii). In pH-responsive orthogonal systems, pH may function as either a gelation trigger or a disruption-based release mechanism, depending on the system, spanning modes (i) and (iii). Recognizing these distinct modes provides a unifying conceptual framework for understanding how pH can be exploited across structurally diverse LMWG systems, and offers a rational basis for the design of next-generation stimuli-responsive soft materials.

Despite their growing significance, these three topics have received comparatively limited coverage in existing reviews relative to more extensively discussed categories, such as peptide- or sugar-based gelators. This review aims to consolidate recent findings in these areas and offer a critical assessment of the progress achieved and challenges remaining.

## 2. N-Oxide-Type Low-Molecular-Weight Hydrogelators

**Figure 2:**
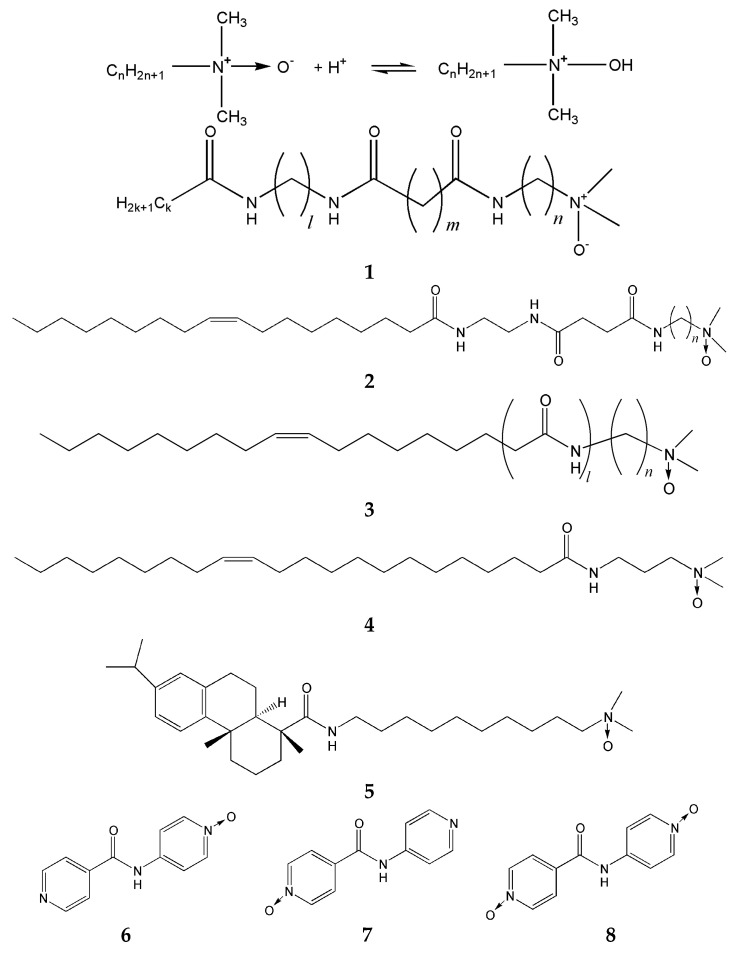

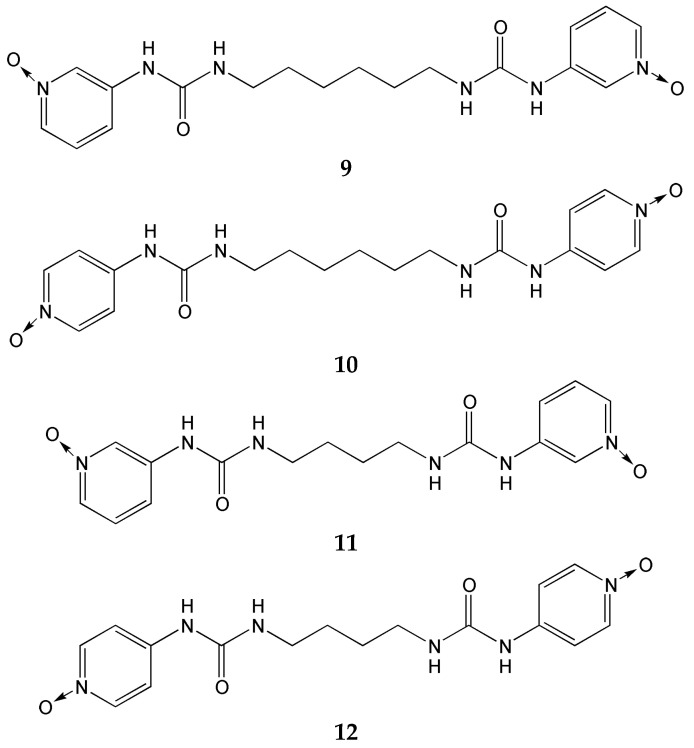
Equilibrium equation of long-chain alkyldimethylamine oxide. (**1**–**5**) chemical structures of amide amine oxide type gelators [27,28,29,30,31,32], (**6**–**8**) chemical structures of N-oxide amide gelators [33,34], (**9**–**12**) chemical structures of pyridyl N-oxide type gelators [35].

N-oxides are weak bases that exist in a nonionic form at high pH and become cationic through protonation at low pH. They also represent functional groups known to enhance water solubility. N-oxides are widely distributed in nature and have been increasingly applied across a range of drug discovery-related contexts, including antibacterial agents and MRI contrast reagents. In this chapter, we introduce two classes of low-molecular-weight gelators incorporating N-oxide moieties that have recently attracted growing attention in biomedical applications: those bearing amine oxide groups, and those based on pyridine-N-oxide frameworks. Surfactants containing amine oxide groups can serve as effective LMWHGs through appropriate control of their chemical structures. For instance, simple long-chain alkyldimethylamine oxide (LAO) surfactants are widely used in dishwashing detergents. LAOs are weakly basic and function as nonionic surfactants at high pH but convert to cationic surfactants at low pH when the amine oxide group becomes protonated (Figure 2). Depending on hydrocarbon chain length and solution pH, LAOs form spherical micelles, worm-like micelles, or vesicles [51,52].

Amidated amine oxide-based surfactants, generated by introducing amide groups into LAOs, function as LMWHGs [27,28,29,30,31,32]. These gelators (labeled **1**–**5** in Figure 2) are characterized by amine oxide and amide groups, both functioning as hydrogen bonding sites. Hydrogen bonding at the amine oxide groups is pH-dependent and partly determines the structural stability of the assemblies and gel formation.

The hydrophobic portions of these molecules adopt different structures. Among the long-chain hydrocarbon chains are long-chain unsaturated hydrocarbons (gelators **2**–**4** in Figure 2) [27,29] and long-chain saturated hydrocarbons (gelator **1** in Figure 2) [28,31,32].

Rosin-derived hydrophobic segments, not typically classified as conventional surfactants, also act as hydrogelators (gelator **5**) [30]. Molecules with long-chain unsaturated hydrocarbons (C18 oleyl; gelators **2** and **3**, C22 erucamide; gelator **4**) and rosin-derived hydrophobic segments exhibit sufficient gelation capabilities despite possessing a single amide group. In contrast, long-chain saturated hydrocarbons with alkyl-chain lengths of C9, C11, and C13 (gelator **1**) are too short for effective hydrophobic interactions, so must rely on hydrogen bonding between multiple amide groups for gelation. This suggests that hydrophobic interactions among hydrocarbon chains synergize with intermolecular hydrogen bonding between the amide groups during hydrogel formation [28,31,32].

**Figure 3:**
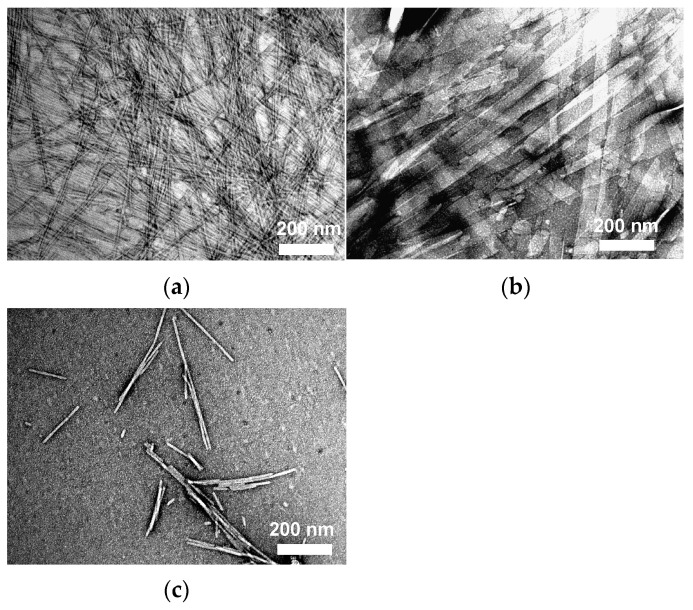
Negative-staining TEM images showing the pH-dependent morphological transition of amidoamine oxide (13-2-2-5) aggregates in water at room temperature. Typical images of (**a**) β = 0, (**b**) β = 0.5, and (**c**) β = 1. Reproduced with permission from [32].

Furthermore, the aggregate morphology correlates with the hydrophobic structure: long-chain unsaturated hydrocarbons (C18 oleyl and C22 erucamide) form worm-like aggregates, whereas molecules with sufficiently long alkyl chains and rigid rosin-derived hydrophobic groups form low-curvature nanobelt (ribbon)-like aggregates [28,30,31,32]. Beyond the influence of molecular structure, aggregate morphology can also be controlled by pH. In a study on amidoamine oxide-based gelators, negative-staining transmission electron microscopy (TEM) observations revealed that the degree of protonation of the amine oxide group governs the morphological transition between aggregate structures: rod-like aggregates are favored at degrees of protonation (β) of 0 and 1, whereas ribbon-like aggregates predominate at β = 0.5 as shown in Figure 3 [32]. This morphological dependence can be rationalized in terms of the distinct intermolecular interactions active at each degree of protonation. At β = 0, the amine oxide groups remain nonionic, and hydrogen bonding between them is effectively absent. At β = 1, the amine oxide groups carry a full positive charge, giving rise to electrostatic repulsion between head groups. In both cases, the suppression of attractive intermolecular interactions at the amine oxide moiety results in the formation of rod-like aggregates with high aggregate curvature. In contrast, at β = 0.5, hydrogen bonding between partially protonated and neutral amine oxide groups becomes effective, reducing the aggregate curvature and thereby promoting the formation of ribbon-like aggregates with a low-curvature morphology. These results demonstrate that pH-controlled modulation of the protonation state provides a rational means of tuning aggregate geometry in amide amine oxide-type LMWHGs, highlighting that pH serves not only as a trigger for sol–gel transitions but also as a tool for fine-tuning the nanoscale architecture of the self-assembled network.

The hydrophobic chain structure, number and arrangement of amide groups, and spacer length of amine-oxide-type gelators can be rationally designed to alter the strength and directionality of their intermolecular interactions. On the downside, synthetic amine oxide-type gelators may be less biocompatible and biodegradable than sugar-based or amino acid-based gelators, which can be safely added to medical preparations, food, and cosmetics and are easily sourced from natural resources with fewer synthetic steps. In addition, sugar-based, amino acid-based, and peptide-based gelators have multipoint hydrogen bonding via the hydroxyl and carboxyl groups. Peptide-based systems, in particular, commonly display self-healing and self-assembly properties [5,8,14]. However, most sugar and amino acid-based gelators must be added at higher concentration (≥1–5 wt%) than amine oxide-based gelators. Furthermore, the natural scaffold limits the freedom of molecular design. Although some amino acid-based gelators respond to pH, many lack the clear reversibility of amine oxide-based gelators. Therefore, sugar and amino acid-based gelators are suitable for medicines, food, cosmetics, and other applications requiring high safety and biodegradability, or when simple preparation and natural origin are important. Conversely, amine oxide-based gelators with superior pH/temperature responsiveness and structural controllability are suitable for functional materials and stimuli-responsive gels. Amine oxide-based gelators that form worm-like micelles, rod-like assemblies, or nanobelt (ribbon)-like assemblies are particularly advantageous for applications demanding high-viscosity, low-concentration gels [27,28,29,30,31,32].

Ghosh et al. synthesized 2 mono-N-oxides (gelators **6** and **7** in Figure 2) and a di-N-oxide (diNO; gelator **8** in Figure 2) by modifying the pyridyl groups of N-(4-pyridyl) isonicotinamide (4PINA), a representative hydrogelator, to N-oxides, and investigated their gelation performance [33]. 4PINA forms one-dimensional fibers through N–H···N interactions, resulting in the formation of robust gels. N-oxidation of 4PINA is expected to increase the hydrophilicity of the molecule. The two mono-N-oxides underwent crystallization, and only diNO, in which both terminal groups were converted to N-oxides, formed a hydrogel. However, the diNO gel exhibited lower mechanical strength and thermal stability compared to the 4PINA gel. Scanning electron microscopy (SEM) observations revealed that the diNO gel formed a needle-like microcrystalline fibrous network, which differed from the efficient fibrous morphology observed in 4PINA. Furthermore, single-crystal X-ray structural analysis indicated that diNO undergoes dimer formation through N–H···O hydrogen bonding and π–π stacking. These results suggest that gel formation in 4PINA is primarily governed by N–H···N hydrogen bonding, whereas in diNO, N–H···N interactions are weakened and C–H···O interactions instead contribute to gel formation. The lower stability of the diNO gel network is attributed to its reliance on relatively weak C–H···O interactions compared to N–H···N hydrogen bonding. In the case of the mono-N-oxides, the increase in hydrophilicity due to hydration may have reduced their gelation ability. In other words, N-oxidation enhances water solubility and crystallinity but may simultaneously compromise gelation performance. For the design of mechanically strong gels, it is effective to exploit linear and unidirectional hydrogen bonding interactions such as N–H···N. On the other hand, the introduction of N-oxidation is also promising for the development of functional materials that leverage stimuli-responsiveness and recrystallization properties.

Ghosh et al. reported that mono-N-oxide pyridyl amides (gelators **6** and **7** in Figure 2) behave as non-gelators in water but form stable metallogels in the presence of ZnCl_2_ or CdCl_2_ [34]. The anion-responsive behavior of these metallogels depends strongly on the nature of the coordinated metal ion: while the Zn^2+^ gels showed responses to multiple anions including CN^−^, F^−^, and I^−^, the Cd^2+^ gels selectively recognized CN^−^ via a gel-to-sol transition, leaving the network intact in the presence of halide ions. This selectivity arises because CN^−^, as a stronger ligand than halides, competitively scavenges Cd^2+^ from the gel matrix, thereby disrupting the metallogel network. These results demonstrate that the type of metal ion incorporated into the N-oxide-based metallogel governs the selectivity toward anionic analytes, suggesting potential application in the visual detection of cyanide in water. However, since gelation strictly requires the presence of metal ions, the applicable conditions are inherently limited, and interference from high concentrations of iodide ions has also been noted for the Cd^2+^ gels.

Sudhakaran Jayabhavan et al. synthesized bis(pyridyl-N-oxide urea) compounds (gelators **9**–**12** in Figure 2) by modifying the 3-pyridyl or 4-pyridyl groups of bis(pyridyl urea) compounds to the corresponding N-oxides, and evaluated their gelation abilities [35]. It is noteworthy that N-oxidation induced hydrogelation in the 3-pyridyl-based compounds (gelators **9** and **11**), whereas the parent bis(pyridyl urea) compounds were insoluble in water and did not form hydrogels. The stimuli-responsive behavior of these hydrogels in the presence of various salts was investigated by rheological measurements. In the presence of K, Na, Mg, and Ca salts, the gel strength of the modified compounds was enhanced, and gelation was induced even below the minimum gel concentration, demonstrating salt-induced gelation. In contrast, the gel network collapsed in the presence of chloride salts of Al(III), Zn(II), Cu(II), and Cd(II). Thus, these N-oxide gelators exhibit a making-and-breaking ability in response to specific metal ions and anions, which is of considerable interest for sensing applications. However, certain salts capable of collapsing the gel network may act as undesirable interferents, and the requirement for specific salt conditions to achieve optimal gel strength may limit the range of applicable environments.

## 3. Boronic Acid Derivatives as Low-Molecular-Weight Hydrogelators

Numerous examples of gelators in which boronic acid, a low-molecular-weight compound, has been covalently incorporated into polymer backbones have been reported, and several review articles have been published on this topic [54,55,56,57]. The majority of these systems exploit the ability of boronic acid to act as a dynamic crosslinking point in response to external stimuli, such as changes in pH or the presence of diol-containing species derived from saccharides. This dynamic crosslinking character strongly contributes to the self-healing properties of the resulting gels, and it is precisely this feature that has driven the progress of research into the incorporation of boronic acid derivatives into polymers for gelator development. Indeed, the concept of dynamic crosslinking is considered highly valuable for the development of self-healing gels.

Even within approximately the last decade, examples have emerged of two-component polymer gelators featuring dynamic crosslinks, in which boronic acid derivatives are incorporated into one polymer component and saccharides or other diol-bearing moieties into the other [58,59,60,61,62,63,64,65,66]. Single-component polymer gelators in which both boronic acid and diol functionalities are incorporated within the same polymer chain have also been reported [67,68,69,70]. Furthermore, approaches aimed at enhancing gel performance by combining multiple polymers in boronic acid-crosslinked systems have been demonstrated [71]. Research in this area has focused primarily on synthetic strategies for introducing boronic acid derivatives into polymer frameworks and on the control of stimuli-responsive behavior. In particular, precise control over stimuli-responsiveness is considered a critically important technology for translating these materials toward industrial applications. It should be noted, however, that all the systems described above are polymeric hydrogelators and do not fall within the category of LMWGs.

**Figure 4:**
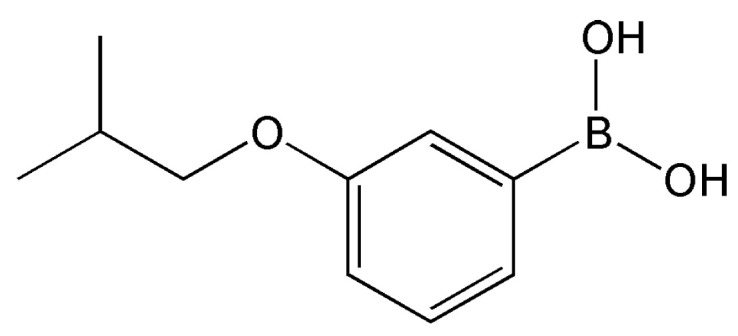
Chemical structure of 3-isobutoxyphenylboronic acid [53].

Indeed, the vast majority of boronic acid-based gelators reported to date are polymeric in nature, and until recently, examples of LMWGs composed of boronic acid derivatives were virtually absent from the literature. In 2023, Orságh and co-workers reported a study in which boronic acid itself functions as an LMWG [53]. Specifically, a single low-molecular-weight compound, 3-isobutoxyphenylboronic acid (Figure 4), was shown to self-assemble in water to form a nanofiber network, thereby producing a hydrogel. In systems where boronic acid is covalently incorporated into a polymer, the reversible boronic ester bond formed between the boronic acid and diol moieties typically serves as the dynamic crosslinking point. In contrast, in this report, hydrogen bonding is identified as the primary driving force for self-assembly and gelation, with no involvement of boronic ester bond-based crosslinking. The hydrogel is formed not through a polymer network, but rather through the physical entanglement of crystalline and amorphous fibers arising from the self-assembly of the low-molecular-weight compound. This work has been presented as the first example of phenylboronic acid alone functioning as an LMWG. In this system, the pH-responsiveness of boronic acid, as well as its responsiveness to H_2_O_2_ and cyclodextrin, are not involved in the gelation mechanism itself, but are instead exploited to achieve stimuli-responsive gel disruption.

It should be emphasized that the use of phenylboronic acid as a standalone LMWG is currently represented by a single reported example, and this field is therefore still in its infancy. Whether 3-isobutoxyphenylboronic acid represents an isolated case or the forerunner of a broader class of boronic acid-based LMWGs remains to be established through future investigation. Nevertheless, the conceptual significance of this finding should not be understated: it demonstrates that hydrogen bonding alone, without boronic ester crosslinking, can serve as the primary driving force for gelation in a low-molecular-weight boronic acid compound. This inversion of the conventional design logic opens a previously unexplored avenue for LMWG development. Future work directed at the systematic exploration of structural analogues, the elucidation of the precise self-assembly mechanism, and the demonstration of practical utility in sensing or controlled release applications will be essential for establishing phenylboronic acid derivatives as a viable class of LMWGs.

## 4. pH-Responsive Orthogonal Self-Assembly Systems

In nature, highly functional structures formed by the self-assembly of lipids and proteins—such as the cytoskeleton, liposomes, and lamellar membranes in biological cells—coexist as distinct supramolecular architectures. Each component retains its own unique properties while coexisting with others, thereby collectively giving rise to a broad repertoire of biological functions that would be unattainable in single-component systems. According to van Esch and co-workers, an orthogonal self-assembling system is defined as one in which different supramolecular structures form independently within a single system, each maintaining its inherent characteristics [76]. From a biomimetic perspective, such systems are of considerable significance, as they aim to reproduce in artificial settings the sophisticated multifunctionality observed in living systems. In practice, however, complete independence between components is extremely difficult to achieve; experimental studies have consistently demonstrated that the constituent assemblies subtly influence one another. Accordingly, truly complete orthogonality should be regarded as an idealized target rather than a routinely attainable outcome.

Over the past decade, a growing number of studies have reported on the control of orthogonal self-assembly in response to pH [72,73,74,75]. Representative examples include combinations of pH-activated low-molecular-weight gelators (LMWGs) and polymer gels [73], hybrid systems comprising pH-responsive LMWGs and polymer gelators (PGs) [74], pH-triggered self-sorting between two distinct LMWGs [72], and orthogonal systems composed of cellulose nanocrystals and an LMWG [75].

**Figure 5:**
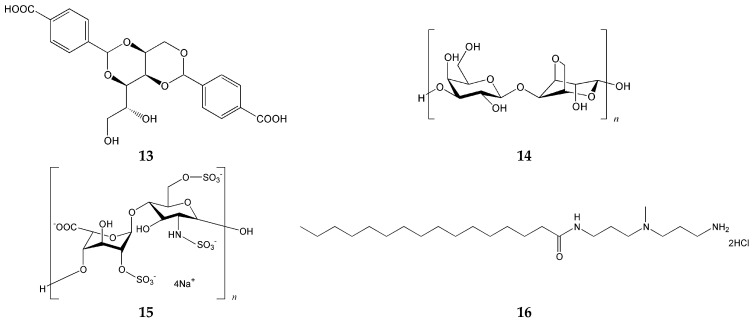
Chemical structures of components incorporated into the multicomponent hybrid hydrogel [73].

Vieira et al. [73] reported a multicomponent hybrid hydrogel consisting of four components: the pH-activated LMWG 1,3;2,4-dibenzylidenesorbitol-4′,4″-dicarboxylic acid (DBS–COOH; gelator **13** in Figure 5), a thermally activated polymer gelator (PG) agarose (gelator **14**), the anionic biopolymer heparin (polymer **15**), and cationic self-assembled multivalent (SAMul; surfactant **16**) micelles capable of binding heparin. This system was designed to achieve the controlled release of heparin. Because DBS–COOH is pH-sensitive, it imparts pH responsiveness to the hybrid material, whereas agarose as the PG provides mechanical robustness. Thus, the two gelator components fulfill distinct functional roles within the material. SAMul serves as a heparin-binding agent and contributes to the inhibition of heparin release. Collectively, these results demonstrate that a multicomponent approach enables exceptionally precise control over the properties of self-assembled materials. Nevertheless, certain limitations must be noted: direct mixing of SAMul with DBS–COOH leads to gel disruption, restricting the range of components that can be incorporated into the system. In addition, stable gel formation requires several hours, which may present a practical challenge for applications that demand rapid gelation.

**Figure 6:**
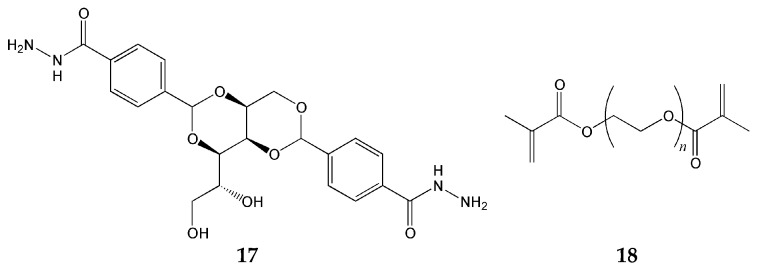
Chemical structures of components of hybrid hydrogel [74].

Chivers et al. employed DBS-CONHNH_2_ (gelator **17** in Figure 6) (DBS = 1,3;2,4-dibenzylidene-D-sorbitol) as the LMWG and poly(ethylene glycol) dimethacrylate (PEGDM; gelator **18**) as the crosslinked polymer gelator (PG) [74]. By applying entirely different gelation triggers—ultrasonication followed by heating and cooling cycles for DBS-CONHNH_2_, and UV photopolymerization for PEGDM—independent network formation was achieved without mutual interference between the two components. This demonstrates the general principle that, when orthogonal triggers are used, each component can assemble without disrupting the self-assembly process of the other. Notably, the pH responsiveness of DBS-CONHNH_2_ functions not as a gelation trigger, but as a means of controlling drug release. By utilizing the pH gradient between the stomach (pH 2) and the intestine (pH 7–8), pH-selective oral drug delivery becomes possible; furthermore, considering that the pH of the skin surface ranges from 5 to 6, this material also demonstrates potential for transdermal administration. However, two limitations should be noted. First, increasing the PEGDM content gradually reduces orthogonality, resulting in interference between networks that is subtle but measurable. Second, because the diffusion of PEGDM into the preformed LMWG network takes time, gel preparation requires approximately three days, which poses a constraint for practical application.

**Figure 7:**
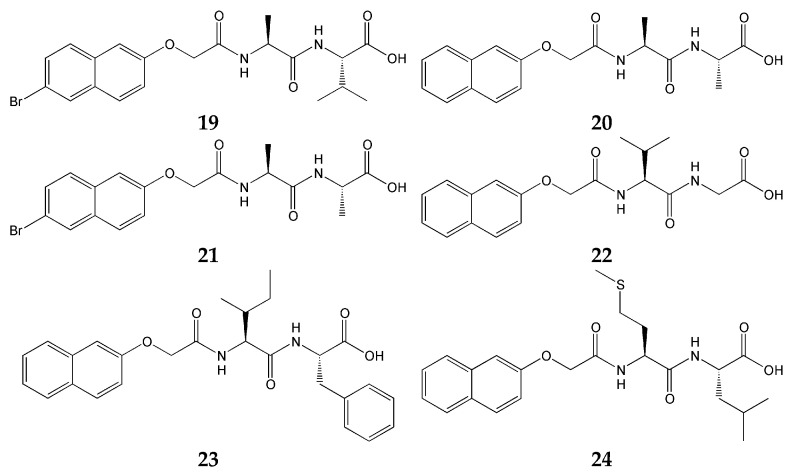
Chemical structures of components of orthogonal systems [72].

In contrast to the two studies discussed above, the work of Colquhoun et al. [72] employs pH responsiveness directly as the gelation trigger. A key feature of their approach is the sequential gelation of two Fmoc-dipeptide-based LMWGs driven by a gradual decrease in pH—from approximately pH 10.5 to pH 4—induced by the hydrolysis of glucono-δ-lactone (GdL). By exploiting differences in the apparent pKa values of the two LMWGs, the authors demonstrated a range of distinct assembly behaviors, including orthogonal self-sorting, delayed gelation, disruptive assembly, and co-assembly. The pKa values of the individual gelators (Figure 7) were reported as follows: 5.9 for gelator **19**, 5.0 for gelator **20**, 5.3 for gelator **21**, 4.5 for gelator **22**, 6.4 for gelator **23**, and 5.9 for gelator **24** [72], and orthogonal self-sorting was confirmed for the combinations **19** + **20**, **21** + **22**, **23** + **24**, and **19** + **21**. It is particularly noteworthy that pH-controlled orthogonal assembly was achieved using only two LMWGs, without recourse to any polymeric component.

Nevertheless, certain limitations remain. Because gelation kinetics are governed by the rate of pH change, rapid gel formation is inherently difficult to achieve with this approach. In addition, the use of D_2_O in place of H_2_O, which is required for characterization purposes, introduces practical disadvantages that may limit the applicability of this system. Furthermore, co-assembly has been attributed to the structural similarity between the two LMWG molecules; however, the precise degree of structural resemblance required to induce co-assembly rather than self-sorting has yet to be established. A clearer understanding of this structure–behavior relationship would be of considerable value for the rational design of multicomponent LMWG systems.

**Figure 8:**
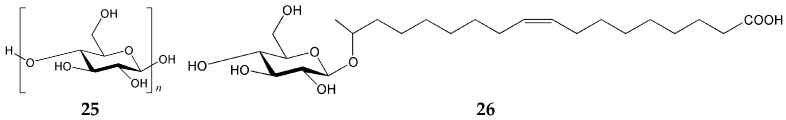
Chemical structures of components of orthogonal systems [75].

Finally, we introduce a very recently reported orthogonal system composed of cellulose (polymer **25** in Figure 8) nanocrystals and an LMWG [75]. In this system, pH responsiveness functions as an indirect gelation trigger. Specifically, the monounsaturated glucolipid G-C18:1 (gelator **26** in Figure 8) forms micelles at alkaline pH, and subsequent addition of Ca^2+^ induces fiber gel formation. The same compound assembles into vesicles in the pH range of 4–6.2 and forms a lamellar precipitate below pH 4. Consequently, a decrease in pH leads to gel collapse, whereas restoration of alkaline conditions recovers the fibrillar network and regenerates gel elasticity. An additional strength of this system lies in the construction of an orthogonal hydrogel from entirely bio-based materials—a feature that aligns well with growing interest in sustainable soft matter. The relatively rapid gel formation is also a practical advantage.

However, several limitations merit attention. Although sulfated cellulose nanocrystals (SCNCs), the second network-forming component, retain their gel state at pH 5 when present alone, the combined G-C18:1/SCNC system undergoes complete gel collapse at the same pH. This indicates that the pH responsiveness of the G-C18:1 network dominates the macroscopic behavior of the entire orthogonal system. From the perspective of controlled drug release, this all-or-nothing response—in which the entire gel disintegrates upon a pH change—may be considered a significant limitation compared with systems that afford more graded or spatially selective release. Furthermore, gelation of G-C18:1 requires not only pH adjustment to alkaline conditions but also the addition of Ca^2+^ ions, meaning that two distinct triggers must be applied simultaneously. This dual-trigger requirement adds complexity to the formulation process and may constrain practical applicability.

Taken together, the three pH-responsive orthogonal systems discussed above share a common conceptual framework but differ markedly in their design strategies, gelation triggers, and practical constraints. In the system reported by Vieira et al., pH responsiveness is conferred by the LMWG component (DBS–COOH), while mechanical robustness is provided by the thermally activated polymer gelator agarose; the principal limitation lies in the incompatibility between certain components upon direct mixing and in the slow gelation kinetics. The system developed by Chivers et al. achieves orthogonality through independent triggers—ultrasonication/thermal cycling for the LMWG and UV photopolymerization for the polymer network—with pH serving not as a gelation trigger but as a means of controlling drug release; however, this system requires approximately three days for preparation, which represents a significant practical limitation. The most recently reported system by Phi et al., composed entirely of bio-based materials, offers the advantage of rapid gel formation and environmental sustainability, but exhibits an all-or-nothing pH response in which the entire gel disintegrates upon acidification, limiting its utility for graded or spatially selective drug release. A further point of distinction concerns the degree of true orthogonality achieved: in all three systems, subtle inter-network interactions were detected, confirming that complete independence between co-assembled structures remains an idealized rather than a practical target. These comparative observations suggest that the rational design of pH-responsive orthogonal systems requires not only the careful matching of gelation triggers and component compatibilities, but also a deliberate consideration of the trade-off between stimulus sensitivity and mechanical integrity.

The structural features, gelation properties, key driving forces, advantages, and limitations of all pH-responsive LMWG systems discussed in this review are summarized in Table 1 and Table 2. These tables are intended to facilitate cross-system comparison and to highlight the recurring trade-offs between stimulus sensitivity, mechanical robustness, and practical applicability that emerge across the three classes of gelators examined here.

## 5. Conclusions

This review has examined three categories of pH-responsive low-molecular-weight gelators (LMWGs) that have shown notable progress over the past decade yet remain relatively underexplored in the literature: N-oxide-type hydrogelators, phenylboronic acid-based LMWGs, and pH-responsive orthogonal self-assembly systems. As summarized in Table 1 and Table 2, these systems differ markedly in their molecular design strategies, the role of pH, and their practical constraints, yet share a common reliance on the interplay between non-covalent interactions and pH-dependent protonation states.

N-oxide-type hydrogelators represent a structurally versatile and pH/temperature-responsive class of LMWGs. Amide amine oxide-based LMWHGs enable rational tuning of aggregate morphology not only through control of the chemical structure but also through the degree of protonation (i.e., pH), yielding worm-like micelles, rod-like assemblies, or nanobelt (ribbon)-like assemblies. Pyridine-N-oxide-based gelators further extend structural diversity; however, N-oxidation generally enhances water solubility and crystallinity at the expense of gelation performance, as exemplified by the reduced mechanical strength and thermal stability of the diNO gel relative to its parent compound 4PINA. Moreover, several pyridine-N-oxide systems require metal ions as indispensable co-triggers, which restricts the applicable conditions. Compared with sugar-, amino acid-, and peptide-based gelators, amine oxide-based gelators offer superior stimuli-responsiveness and structural tunability, but the challenge of reconciling functional sophistication with biological safety remains an open issue.

The identification of 3-isobutoxyphenylboronic acid as the first example of a phenylboronic acid functioning as an LMWG is conceptually significant. This system demonstrates that hydrogen bonding, rather than boronic ester crosslinking, can serve as the primary driving force for gelation—an inversion of the conventional design logic dominant in polymer-based boronic acid systems. The pH responsiveness, together with responsiveness to H_2_O_2_ and cyclodextrin, is exploited for stimuli-triggered gel disruption rather than gel formation, offering interesting possibilities for controlled release and sensing. Nevertheless, this field remains in its infancy, and whether this compound represents an isolated example or the vanguard of a broader class will require more extensive investigation.

The orthogonal self-assembly approach offers a compelling biomimetic strategy for achieving multifunctionality inaccessible in single-component systems. However, truly complete orthogonality has not been demonstrated in any reported system; subtle inter-network interactions appear to be an inherent feature rather than an exceptional limitation. Practical constraints—including prolonged preparation times, component incompatibility, and the need for multiple simultaneous triggers—continue to hinder translation toward applications. Furthermore, the structural determinants governing self-sorting versus co-assembly in purely LMWG-based systems remain incompletely understood, limiting rational molecular design.

A unifying critical observation across all three topics is that pH responsiveness, while powerful as a design principle, often introduces trade-offs: enhanced stimulus sensitivity may compromise mechanical robustness, and functional groups conferring pH responsiveness can simultaneously perturb the intermolecular interactions required for effective gelation. The rational integration of computational approaches with experimental molecular design, and the development of biocompatible pH-responsive LMWGs with clinically relevant stimuli thresholds, will be essential for advancing this field toward practical applications in drug delivery, sensing, and responsive soft materials.

## Figures and Tables

**Table 1:** Summary of pH-responsive low-molecular-weight gelators discussed in this review, including their structural features and gelation properties.

Gelator [Reference]	Category	Molecular Structure Feature	pH Responsiveness	Aggregate Morphology
**1**–**5** [27,28,29,30,31,32]	Amide amine oxide	Amide + N-oxide	pH 3–10	Worm-like/rod-like/ribbon-like
**8** (diNO) [33,34]	Pyridine N-oxide	di-N-oxide amide	—	Needle-like fibrous
**6**, **7** [33,34]	Pyridine N-oxide	mono-N-oxide amide	—	—
**9**–**12** [35]	Pyridine N-oxide	bis(pyridyl-N- oxide urea)	Salt-responsive	—
3-isobutoxyphenylboronic acid [53]	Boronic acid	Phenylboronic acid	pH, H_2_O_2_, cyclodextrin	Nanofiber network
DBS–COOH (**13**) + agarose system (**14**) [73]	Orthogonal (LMWG + PG)	Sorbitol-based LMWG	pH-activated LMWG	Fibrous + polymer network
DBS-CONHNH_2_ (**17**) + PEGDM (**18**) [74]	Orthogonal (LMWG + PG)	Sorbitol-based LMWG	pH-controlled release	Fibrous + polymer network
G-C18:1 (**26**) + SCNC (**25**) [75]	Orthogonal (LMWG + nanocrystal)	Glucolipid	pH-triggered collapse	Fibrous + nanocrystal

**Table 2:** Key driving forces, advantages, and limitations of pH-responsive low-molecular-weight gelators discussed in this review.

Gelator [Reference]	Key Driving Force	Advantages	Limitations
**1**–**5** [27,28,29,30,31,32]	Hydrophobic interactions + H-bonding (amide and N-oxide groups)	Clear pH and temperature responsiveness; rational tuning of aggregate morphology; effective at low concentration	Lower biocompatibility and biodegradability than sugar- or amino acid-based gelators
**8** (diNO) [33,34]	H-bonding (C–H···O) + π–π stacking	Water-soluble; stimuli-responsive; potential for functional material applications	Reduced mechanical strength and thermal stability relative to parent compound (4PINA)
**6**, **7** [33,34]	Metal coordination + H-bonding	Selective anion sensing (CN^−^ detection); tunable selectivity depending on metal ion (Zn^2+^ vs. Cd^2+^)	Gelation strictly requires metal ions; susceptible to interference from iodide ions
**9**–**12** [35]	H-bonding (urea groups) + N-oxide···ion interactions	Salt-induced gelation; making-and-breaking ability in response to specific metal ions and anions	Certain salts act as undesirable interferents; optimal gel strength requires specific salt conditions
3-isobutoxyphenylboronic acid [53]	H-bonding (no boronic ester crosslinking)	First example of phenylboronic acid as LMWG; multi-stimuli-responsive; interesting for controlled release and sensing	Single reported example; field still in its infancy; gelation mechanism not yet fully elucidated
DBS–COOH (**13**) + agarose system (**14**) [73]	H-bonding (LMWG) + thermal gelation (polymer)	Precise control over heparin release; complementary functional roles of each component	Direct mixing of SAMul with DBS–COOH causes gel disruption; slow gelation (several hours)
DBS-CONHNH_2_ (**17**) + PEGDM (**18**) [74]	H-bonding (LMWG) + covalent crosslinking via UV photopolymerization (polymer)	Independent network formation via orthogonal triggers; pH-controlled drug release for oral and transdermal delivery	Gel preparation requires approximately three days; orthogonality decreases with increasing PEGDM content
G-C18:1 (**26**) + SCNC (**25**) [75]	H-bonding + hydrophobic interactions (LMWG) + electrostatic interactions (nanocrystal)	Entirely bio-based; rapid gel formation; reversible pH-responsive gel collapse and recovery	All-or-nothing pH response limits graded drug release; gelation requires two simultaneous triggers (alkaline pH + Ca^2+^)

## Data Availability

The data presented in this study are openly available in the article.

## References

[1] Eelkema R., Pich A. (2020). Pros and cons: Supramolecular or macromolecular: What is best for functional hydrogels with advanced properties?. Adv. Mater..

[2] Sangeetha N.M., Maitra U. (2005). Supramolecular gels: Functions and uses. Chem. Soc. Rev..

[3] Shimizu T., Masuda M., Minamikawa H. (2005). Supramolecular nanotube architectures based on amphiphilic molecules. Chem. Rev..

[4] Steed J.W. (2011). Supramolecular gel chemistry: Developments over the last decade. Chem. Commun..

[5] Adams D.J. (2011). Dipeptide and tripeptide conjugates as low-molecular-weight hydrogelators. Macromol. Biosci..

[6] Svobodová H., Noponen V., Kolehmainen E., Sievänen E. (2012). Recent advances in steroidal supramolecular gels. RSC Adv..

[7] Yamanaka M. (2013). Urea derivatives as low-molecular-weight gelators. J. Incl. Phenom. Macrocycl. Chem..

[8] Tomasini C., Castellucci N. (2013). Peptides and peptidomimetics that behave as low molecular weight gelators. Chem. Soc. Rev..

[9] Yi T., Yu X., Chen L., Li Z.T., Whu L.Z. (2015). Hydrogen bonding for the self-assembly of organogels and hydrogels. Hydrogen Bonded Supramolecular Materials.

[10] Du X., Zhou J., Shi J., Xu B. (2015). Supramolecular hydrogelators and hydrogels: From soft matter to molecular biomaterials. Chem. Rev..

[11] Datta S., Bhattacharya S. (2015). Multifarious facets of sugar-derived molecular gels: Molecular features, mechanism of self-assembly and emerging applications. Chem. Soc. Rev..

[12] Wang C., Wiener C.G., Cheng Z., Vogt B.D., Weiss R.A. (2016). Modulation of the mechanical properties of hydrophobically modified supramolecular hydrogels by surfactant-driven structural rearrangement. Macromolecules.

[13] Okesola B.O., Smith D.K. (2016). Applying low-molecular weight supramolecular gelators in an environmental setting-self-assembled gels as smart materials for pollutant removal. Chem. Soc. Rev..

[14] Zengin A. (2016). Enzymatic Degradation of Self-Assembled Peptide Nanofiber Gels. Ph.D. Thesis.

[15] Hanabusa K., Suzuki M. (2016). Physical gelation by low-molecular-weight compounds and development of gelators. Bull. Chem. Soc. Jpn..

[16] Draper E.R., Adams D.J. (2017). Low-molecular-weight gels: The state of the art. Chem.

[17] Chivers P.R.A., Smith D.K. (2019). Shaping and structuring supramolecular gels. Nat. Rev. Mater..

[18] Kumar S., Bajaj A. (2020). Advances in self-assembled injectable hydrogels for cancer therapy. Biomater. Sci..

[19] Panja S., Panja A., Ghosh K. (2021). Supramolecular gels in cyanide sensing: A review. Mater. Chem. Front..

[20] Morris J., Bietsch J., Bashaw K., Wang G. (2021). Recently developed carbohydrate based gelators and their applications. Gels.

[21] Tyagi R., Singh K., Srivastava N., Sagar R. (2023). Recent advances in carbohydrate-based gelators. Mater. Adv..

[22] Aramaki K., Ikeda N. (2023). Formulation of orthogonal hydrogels by surfactant mediated method. Acc. Mater. Surf. Res..

[23] Smith D.K. (2024). Supramolecular gels—A panorama of low-molecular-weight gelators from ancient origins to next-generation technologies. Soft Matter.

[24] Khayat Z. (2023). Recent advances in sensing neutral molecules using low molecular weight gelators (LMWGs). Supramol. Chem..

[25] Choi H., Choi W.S., Jeong J.O. (2024). A review of advanced hydrogel applications for tissue engineering and drug delivery systems as biomaterials. Gels.

[26] Holey S.A., Nayak R.R. (2024). Harnessing glycolipids for supramolecular gelation: A contemporary review. ACS Omega.

[27] Kakehashi R., Tokai N., Yamamura S. (2012). Solution behavior of long–alkyl–chain amide amine oxide surfactants having multiple hydrogen-bonding sites. Chem. Lett..

[28] Kakehashi R., Tokai N., Maeda H. (2015). Effects of the spacer length on the aggregate formation and the gelation of alkylamide amine oxides. Colloid Polym. Sci..

[29] Zhang Y., An P., Liu X. (2015). A “worm”-containing viscoelastic fluid based on single amine oxide surfactant with an unsaturated C 22-tail. RSC Adv..

[30] Chen H., Yan T., Zhang J., Pei X., Cui Z., Song B. (2021). Formation of asymmetric belt-like aggregates from a bio-based surfactant derived from dehydroabietic acid. Soft Matter.

[31] Kakehashi R., Tokai N., Nakagawa M., Kawasaki K., Horiuchi S., Yamamoto A. (2023). Amidoamine oxide surfactants as low-molecular-weight hydrogelators: Effect of methylene chain length on aggregate structure and rheological behavior. Gels.

[32] Kakehashi R., Tokai N., Nakagawa M. (2024). Aggregate Formation and Gelation Behavior of Amidoamine Oxide Surfactants in Water. J. Netw. Polym. Jpn..

[33] Ghosh D., Mulvee M.T., Damodaran K.K. (2019). Tuning gel state properties of supramolecular gels by functional group modification. Molecules.

[34] Ghosh D., Deepa, Damodaran K.K. (2020). Metal complexation induced supramolecular gels for the detection of cyanide in water. Supramol. Chem..

[35] Sudhakaran Jayabhavan S., Ghosh D., Damodaran K.K. (2021). Making and breaking of gels: Stimuli-responsive properties of bis(pyridyl-n-oxide urea) gelators. Molecules.

[36] Marton L., McBain J.W., Vold R.D. (1941). An electron microscope study of curd fibers of sodium laurate. J. Am. Chem. Soc..

[37] Liang J., Ma Y., Zheng Y., Davis H.T., Chang H.T., Binder D., Abbas S., Hsu F.L. (2001). Solvent-induced crystal morphology transformation in a ternary soap system: Sodium stearate crystalline fibers and platelets. Langmuir.

[38] Heppenstall-Butler M., Butler M.F. (2003). Nonequilibrium behavior in the three-component system stearic acid−sodium stearate−water. Langmuir.

[39] Yuan Z., Lu W., Liu W., Hao J. (2008). Gel phase originating from molecular quasi-crystallization and nanofiber growth of sodium laurate–water system. Soft Matter.

[40] Suzuki M., Owa S., Shirai H., Hanabusa K. (2007). Supramolecular hydrogel formed by glucoheptonamide of L-lysine: Simple preparation and excellent hydrogelation ability. Tetrahedron.

[41] Suzuki M., Yumoto M., Kimura M., Shirai H., Hanabusa K. (2003). A family of low-molecular-weight hydrogelators based on L-lysine derivatives with a positively charged terminal group. Chem. Eur. J..

[42] Kiyonaka S., Shinkai S., Hamachi I. (2003). Combinatorial library of low molecular-weight organo-and hydrogelators based on glycosylated amino acid derivatives by solid-phase synthesis. Chem. Eur. J..

[43] Minakuchi N., Hoe K., Yamaki D., Ten-No S., Nakashima K., Goto M., Mizuhata M., Maruyama T. (2012). Versatile supramolecular gelators that can harden water, organic solvents and ionic liquids. Langmuir.

[44] Quigley E., Johnson J., Liyanage W., Nilsson B.L. (2020). Impact of gelation method on thixotropic properties of phenylalanine-derived supramolecular hydrogels. Soft Matter.

[45] Tang C., Smith A.M., Collins R.F., Ulijn R.V., Saiani A. (2009). Fmoc-diphenylalanine self-assembly mechanism induces apparent pKa shifts. Langmuir.

[46] Najafi H., Tamaddon A.M., Abolmaali S., Borandeh S., Azarpira N. (2021). Structural, mechanical, and biological characterization of hierarchical nanofibrous Fmoc-phenylalanine-valine hydrogels for 3D culture of differentiated and mesenchymal stem cells. Soft Matter.

[47] Hoshizawa H., Minemura Y., Yoshikawa K., Suzuki M., Hanabusa K. (2013). Thixotropic hydrogelators based on a cyclo (dipeptide) derivative. Langmuir.

[48] Morita K., Nishimura Y., Ishii J., Maruyama T. (2022). Micelle-like nanoassemblies of short peptides create antimicrobial selectivity in a conventional antifungal drug. ACS Appl. Nano Mater..

[49] Tominaga Y., Kanemitsu S., Yamamoto S., Kimura T., Nishida Y., Morita K., Maruyama T. (2023). Thermally irreversible supramolecular hydrogels record thermal history. Colloids Surf. A Physicochem. Eng. Asp..

[50] Shinde S.D., Kulkarni N., Sahu B. (2023). Synthesis and investigation of backbone modified squaramide dipeptide self-assembly. ACS Appl. Bio Mater..

[51] Maeda H., Kakehashi R. (2000). Effects of protonation on the thermodynamic properties of alkyl dimethylamine oxides. Adv. Colloid Interface Sci..

[52] Kawasaki H., Souda M., Tanaka S., Nemoto N., Karlsson G., Almgren M., Maeda H. (2002). Reversible vesicle formation by changing pH. J. Phys. Chem. B.

[53] Orságh M., Strachota B., Pavlova E., Pánek J., Adamczyk-Woźniak A., Sporzyński A., Leszczyński P., Štěpánek M., Uchman M. (2023). Meta-Isobutoxy Phenylboronic Acid for Nanoscale Multi-Stimuli-Responsive Low-Molecular-Weight Hydrogelator. ACS Appl. Nano Mater..

[54] Cho S., Hwang S.Y., Oh D.X., Park J. (2021). Recent progress in self-healing polymers and hydrogels based on reversible dynamic B–O bonds: Boronic/boronate esters, borax, and benzoxaborole. J. Mater. Chem. A.

[55] Brooks W.L., Sumerlin B.S. (2016). Synthesis and applications of boronic acid-containing polymers: From materials to medicine. Chem. Rev..

[56] Marco-Dufort B., Tibbitt M.W. (2019). Design of moldable hydrogels for biomedical applications using dynamic covalent boronic esters. Mater. Today Chem..

[57] Terriac L., Helesbeux J.J., Maugars Y., Guicheux J., Tibbitt M.W., Delplace V. (2024). Boronate ester hydrogels for biomedical applications: Challenges and opportunities. Chem. Mater..

[58] Sun P., Tian S., Lin M., Chen G. (2018). The glyco-regioisomerism effect on dynamic interactions between glycopolymers with galactose pendants and benzoxaborole-containing polymer. Sci. China Chem..

[59] Chen Y., Wang W., Wu D., Nagao M., Hall D.G., Thundat T., Narain R. (2018). Injectable self-healing zwitterionic hydrogels based on dynamic benzoxaborole–sugar interactions with tunable mechanical properties. Biomacromolecules.

[60] Chen Y., Diaz-Dussan D., Wu D., Wang W., Peng Y.Y., Asha A.B., Hall D.G., Ishihara K., Narain R. (2018). Bioinspired Self-Healing Hydrogel Based on Benzoxaborole-Catechol Dynamic Covalent Chemistry for 3D Cell Encapsulation. ACS Macro Lett..

[61] Li Y., Yang L., Zeng Y., Wu Y., Wei Y., Tao L. (2019). Self-healing hydrogel with a double dynamic network comprising imine and borate ester linkages. Chem. Mater..

[62] Kang H., Wei W., Sun L., Yu R., Yang E., Wu X., Dai H. (2023). Modular design and bonding mechanism of internal boron–nitrogen coordinated boronic ester hydrogels with alkaline pH responsiveness and tunable gelation pH. Chem. Mater..

[63] Marozas I.A., Anseth K.S., Cooper-White J.J. (2019). Adaptable boronate ester hydrogels with tunable viscoelastic spectra to probe timescale dependent mechanotransduction. Biomaterials.

[64] Liu Y., Liu Y., Wang Q., Han Y., Chen H., Tan Y. (2020). Doubly dynamic hydrogel formed by combining boronate ester and acylhydrazone bonds. Polymers.

[65] Yesilyurt V., Webber M.J., Appel E.A., Langer R., Anderson D.G. (2016). Injectable Self-Healing Glucose-Responsive Hydrogels with pH-Regulated Mechanical Properties. Adv. Mater..

[66] Yesilyurt V., Ayoob A.M., Appel E.A., Borenstein J.T., Langer R., Anderson D.G. (2017). Mixed reversible covalent crosslink kinetics enable precise, hierarchical mechanical tuning of hydrogel networks. Adv. Mater..

[67] Dong Y., Wang W., Veiseh O., Appel E.A., Xue K., Webber M.J., Tang B.C., Yang X.-W., Weir G.C., Langer R. (2016). Injectable and glucose-responsive hydrogels based on boronic acid–glucose complexation. Langmuir.

[68] Pettignano A., Grijalvo S., Haering M., Eritja R., Tanchoux N., Quignard F., D’ıaz D.D. (2017). Boronic acid-modified alginate enables direct formation of injectable, self-healing and multistimuli-responsive hydrogels. Chem. Commun..

[69] Hong S.H., Kim S., Park J.P., Shin M., Kim K., Ryu J.H., Lee H. (2018). Dynamic bonds between boronic acid and alginate: Hydrogels with stretchable, self-healing, stimuli-responsive, remoldable, and adhesive properties. Biomacromolecules.

[70] Zhang J., Tanaka J., Gurnani P., Wilson P., Hartlieb M., Perrier S. (2017). Self-Assembly and Disassembly of Stimuli Responsive Tadpole-Like Single Chain Nanoparticles Using a Switchable Hydrophilic/Hydrophobic Boronic Acid Cross-Linker. Polym. Chem..

[71] Liu F., Li G., An Z., Wang S., Xu S., Liu H. (2025). Dynamic boronate ester based hydrogel with enhanced mechanical properties and multi-stimuli-triggered release for tissue repair and antioxidant therapy. Gels.

[72] Colquhoun C., Draper E.R., Eden E.G., Cattoz B.N., Morris K.L., Chen L., McDonald T.O., Terry A.E., Griffiths P.C., Serpell L.C. (2014). The effect of self-sorting and co-assembly on the mechanical properties of low molecular weight hydrogels. Nanoscale.

[73] Vieira V.M., Hay L.L., Smith D.K. (2017). Multi-component hybrid hydrogels–understanding the extent of orthogonal assembly and its impact on controlled release. Chem. Sci..

[74] Chivers P.R., Smith D.K. (2017). Spatially-resolved soft materials for controlled release–hybrid hydrogels combining a robust photo-activated polymer gel with an interactive supramolecular gel. Chem. Sci..

[75] Phi T.L., Kontturi E., Baccile N. (2025). Orthogonality between cellulose nanocrystals and a low-molecular weight gelator. J. Colloid Interface Sci..

[76] van Esch J.H., Feringa B.L. (2000). New functional materials based on self-assembling organogels: From serendipity towards design. Angew. Chem. Int. Ed..

